# Supplementary material for: mTORC2-driven chromatin cGAS mediates chemoresistance through epigenetic reprogramming in colorectal cancer
Source: Nat Cell Biol. 2024 Jul 30;26(9):1585–96. doi: 10.1038/s41556-024-01473-0 (PMC11392818; doi:10.1038/s41556-024-01473-0)
Supplement: Supplementary file 1 — Reporting Summary [file 41556_2024_1473_MOESM1_ESM.pdf]

nature portfolio

Corresponding author(s): Yuan-Lin

Last updated by author(s): Jun 26, 2024

Reporting Summary

Nature Portfolio wishes to improve the reproducibility of the work that we publish. This form provides structure for consistency and transparency in reporting. For further information on Nature Portfolio policies, see our Editorial Policies and the Editorial Policy Checklist.

Statistics

For all statistical analyses, confirm that the following items are present in the figure legend, table legend, main text, or Methods section.

n/a	Confirmed	
☐	☒	The exact sample size (*n*) for each experimental group/condition, given as a discrete number and unit of measurement
☐	☒	A statement on whether measurements were taken from distinct samples or whether the same sample was measured repeatedly
☐	☒	The statistical test(s) used AND whether they are one- or two-sided<br>*Only common tests should be described solely by name; describe more complex techniques in the Methods section.*
☐	☒	A description of all covariates tested
☐	☒	A description of any assumptions or corrections, such as tests of normality and adjustment for multiple comparisons
☐	☒	A full description of the statistical parameters including central tendency (e.g. means) or other basic estimates (e.g. regression coefficient) AND variation (e.g. standard deviation) or associated estimates of uncertainty (e.g. confidence intervals)
☐	☒	For null hypothesis testing, the test statistic (e.g. *F*, *t*, *r*) with confidence intervals, effect sizes, degrees of freedom and *P* value noted<br>*Give P values as exact values whenever suitable.*
☒	☐	For Bayesian analysis, information on the choice of priors and Markov chain Monte Carlo settings
☒	☐	For hierarchical and complex designs, identification of the appropriate level for tests and full reporting of outcomes
☒	☐	Estimates of effect sizes (e.g. Cohen's *d*, Pearson's *r*), indicating how they were calculated

Our web collection on statistics for biologists contains articles on many of the points above.

Software and code

Policy information about availability of computer code

Data collection	MS/MS data were collected by liquid chromatography-tandem mass spectrometry(LC-MS/MS);<br>FACS data was collected by FACScalibur.
Data analysis	MS/MS data were searched against the reviewed UniProt Human protein database (version 20230119 containing 20,308 entries) using Mascot 2.5.1 (Matrix Science), and data analysis was performed using Scaffold 4.4.8 software (Proteome Software);<br>FACS data analysis was performed with FlowJo software;<br>All statistical analyses were performed with GraphPad Prism 9 and SPSS 19.0 software.

For manuscripts utilizing custom algorithms or software that are central to the research but not yet described in published literature, software must be made available to editors and reviewers. We strongly encourage code deposition in a community repository (e.g. GitHub). See the Nature Portfolio guidelines for submitting code & software for further information.

Data

Policy information about availability of data

All manuscripts must include a data availability statement. This statement should provide the following information, where applicable:

- Accession codes, unique identifiers, or web links for publicly available datasets
- A description of any restrictions on data availability
- For clinical datasets or third party data, please ensure that the statement adheres to our policy

All data generated or analyzed during this study are included in this article and its Supplementary Information.

Research involving human participants, their data, or biological material

Policy information about studies with human participants or human data. See also policy information about sex, gender (identity/presentation), and sexual orientation and race, ethnicity and racism.

Reporting on sex and gender	Tumor specimens were collected from 6 colorectal cancer males patients (ages 40-60 years) undergoing surgical resection at the Peking University Shenzhen Hospital.
Reporting on race, ethnicity, or other socially relevant groupings	Asian
Population characteristics	All patients had previously received 5-FU chemotherapy.
Recruitment	Patients did not receive compensation for providing samples.
Ethics oversight	Related experiments were approved by the Institutional Human Research Ethics and Animal Care and Use Committee at Peking University and Peking University Shenzhen Hospital.

Note that full information on the approval of the study protocol must also be provided in the manuscript.

Field-specific reporting

Please select the one below that is the best fit for your research. If you are not sure, read the appropriate sections before making your selection.

☒ Life sciences ☐ Behavioural & social sciences ☐ Ecological, evolutionary & environmental sciences

For a reference copy of the document with all sections, see nature.com/documents/nr-reporting-summary-flat.pdf

Life sciences study design

All studies must disclose on these points even when the disclosure is negative.

Sample size	Data of most experiments were collected from at least 3 independent samples.
Data exclusions	No data were excluded.
Replication	All attempts at replication were successful.
Randomization	After the establishment of Cgas-KO mouse model, the animals were randomly grouped for in vivo experiments.
Blinding	The investigator was blinded for in vivo experiments of all animal experiments.

Reporting for specific materials, systems and methods

We require information from authors about some types of materials, experimental systems and methods used in many studies. Here, indicate whether each material, system or method listed is relevant to your study. If you are not sure if a list item applies to your research, read the appropriate section before selecting a response.

Materials & experimental systems

n/a	Involved in the study
☐	☒ Antibodies
☐	☒ Eukaryotic cell lines
☒	☐ Palaeontology and archaeology
☐	☒ Animals and other organisms
☒	☐ Clinical data
☒	☐ Dual use research of concern
☒	☐ Plants

Methods

n/a	Involved in the study
☒	☐ ChIP-seq
☐	☒ Flow cytometry
☒	☐ MRI-based neuroimaging

Antibodies

Antibodies used	Antibodies used in immunoblotting: Anti-cGAS (CST, #15102, D1D3G, 1:1,000 dilution), anti-HELLS (CST, #7998, 1:1,000), anti-β-Actin (CST, #4967, 1:1,000), anti-PDI (CST, #2446, 1:1,000), anti-HA (CST, #3724, C29F4, 1:1,000), anti-Flag (CST, #14793, D6W5B, 1:1,000), anti-Histone H3 (CST, #9715, 1:1000), anti-ARID1A (CST, #12354, D2A8U, 1:1000), anti-MCM7 (CST, #3735, D10A11, 1:1000), anti-SMARCC2 (CST, #12760, D8O9V, 1:1000), anti-Akt (CST, #9272, 1:1,000), anti-pSer473-Akt (CST, #4060, D9E, 1:1,000), anti-MCM3 (CST, #4012, 1:1,000), anti-GLS1 (CST, #49363, E4T9Q, 1:1,000), anti-mTOR (CST, #2983, 7C10, 1:1,000), anti-RICTOR (CST, #2114, 53A2, 1:1,000), anti-RAPTOR (CST, #48648, E6O3A, 1:1,000), anti-SIN1 (CST, #12860, D7G1A, 1:1,000), anti-PAI-1 (CST, #11907, D9C4, 1:1,000), anti-BAF200 (CST, #82342, D8D8U, 1:1,000), anti-CDC45(CST, #11881, D7G6, 1:1,000), anti-p70 S6K (CST, #9202, 1:1,000), anti-pThr389-p70 S6K (CST, #9209, 1:1,000), anti-4E-BP1 (CST, #9452, 1:1,000), anti-pSer65-4E-BP1 (CST, #9451, 1:1,000), and anti-GAPDH (CST, #5174, D16H11, 1:2,000) were purchased from Cell Signaling Technology. Anti-KGA (Proteintech, 20170-1-AP, 1:1,000) and anti-GAC (Invitrogen, PA5-40134, 1:1,000) were purchased from Thermo Fisher Scientific. Anti-SMARCA4 (Sigma, MABE121, 1:1000), anti-MCM5 (Sigma, SAB1406111, 1:1,000) were purchased from Sigma-Aldrich.<br>Antibodies used in immunoprecipitation and immunofluorescence: Anti-cGAS (CST, #79978, E5V3W, IF, 1:200), anti-cGAS (CST, #31659, D3O8O, IP, 1:200), anti-HA (CST, #3724, C29F4, 1:200), anti-H2A (CST, #12349, D6O3A, 1:200), and Phalloidin (CST, #8953, 1:200) were purchased from Cell Signaling Technology. The polyclonal anti-pSer37-cGAS antibodies generated by ourselves were derived from rabbits. The antigen sequence used for immunization was cGAS aa29-47 (GAPMDPTES*PAAPEAALPK). S* stands for phosphorylated serine residue in these synthetic peptides. The antibodies were affinity purified using the antigen peptide column, but they were not counter selected on unmodified antigen.
Validation	The purchased antibodies were all validated by the manufactures in their specific data sheets.

Eukaryotic cell lines

Policy information about cell lines and Sex and Gender in Research

Cell line source(s)	HCT116, HT29, SW480, MC38, HEK293T, MDA-MB-231, 786-0, HCC44 cells were purchased from ATCC
Authentication	Cell lines were authenticated by STR profiling.
Mycoplasma contamination	Cell lines are regularly screened to ensure the absence of mycoplasma contamination using MycoAlert Mycoplasma detection kit (lonza).
Commonly misidentified lines<br>(See ICLAC register)	No commonly misidentified cell lines were used.

Animals and other research organisms

Policy information about studies involving animals; ARRIVE guidelines recommended for reporting animal research, and Sex and Gender in Research

Laboratory animals	6 weeks old BALB/c nude mice were used in this study
Wild animals	Wild animals are not involved in this study.
Reporting on sex	Findings apply to both sex of animals, there is no sex distinction in this study.
Field-collected samples	None used
Ethics oversight	All animal experiments were conducted in accordance with the 'Guide for the Care and Use of Laboratory Animals' and 'Principles for the Utilization and Care of Vertebrate Animals', and were approved by the Institutional Human Research Ethics and Animal Care and Use Committee at Peking University and Peking University Shenzhen Hospital. Mice were monitored daily and experiments were terminated if tumor diameters exceeded 15 mm or volumes exceeded 2,000 mm3, in accordance with guidelines established by our Institutional Animal Care and Use Committee to prevent unnecessary suffering.

Note that full information on the approval of the study protocol must also be provided in the manuscript.

Plants

Seed stocks

Seed stocks are not involved in this study.

Novel plant genotypes

Novel plant genotypes are not involved in this study.

Authentication

not involved in this study.

Flow Cytometry

Plots

Confirm that:

☒ The axis labels state the marker and fluorochrome used (e.g. CD4-FITC).

☒ The axis scales are clearly visible. Include numbers along axes only for bottom left plot of group (a 'group' is an analysis of identical markers).

☒ All plots are contour plots with outliers or pseudocolor plots.

☒ A numerical value for number of cells or percentage (with statistics) is provided.

Methodology

Sample preparation

Cells were incubated with 33 µM BrdU for 20 min, collected and centrifuged at 250G for 10 min. The pellet was re-suspended in 750µL of PBS 1X and fixed by adding 2250µL of ice-cold (−20°C) pure ethanol dropwise while vortexing. Samples were washed once in 1%BSA/PBS and re-suspended in 1 mL of 2N HCl and incubated for 25 min at room temperature allowing DNA denaturation. Then 3mL of 0.1M Sodium Borate (pH 8.5) was added to neutralize the acidic pH of the HCl solution and samples were incubated at room temperature for 2 min, centrifuged and washed twice in 1%BSA/PBS. Samples were then transferred in Eppendorf tube and centrifuged at 800G for 5 min. Pellets were re-suspended in 100µL of pure anti-BrdU antibody (life-technologies) diluted 1:5 in 1%BSA/PBS and incubated for 1 h at room temperature in the dark. Samples were washed with 1%BSA/PBS and re-suspended in 100µL of anti-mouse FITC (life-technologies) diluted 1:50 in 1%BSA/PBS for 1 h at room temperature in the dark. After washing once with 1%BSA/PBS pellets were re-suspended in 1 mL of Propidium Iodate (PI) (2.5 µg/mL) and RNase (250 µg/mL) (RibonucleaseA from bovine pancreas, Sigma) and incubated overnight at 4°C.

Instrument

FACScalibur

Software

FlowJo software

Cell population abundance

Cell population abundance is not involved in this study

Gating strategy

The gating strategy for cell cycle analysis by flow cytometry:
1.Forward scatter area (FSC-A) vs Side scatter area (SSC-A) - Gate on the main cell population to exclude debris and clumps.
2.Doublet discrimination - Gate on single cells by plotting FSC-Width vs FSC-Area/Height. This will exclude doublets or clusters of cells.
3.DNA content - Plot the DNA-intercalating fluorescent dye (e.g. propidium iodide, DAPI) on the y-axis. This allows visualization of cells in G0/G1, S, and G2/M phases.
4.G0/G1 peak - Draw a gate around the first peak/largest population of cells with 2N DNA content (G0/G1 phase).
5.S phase - Identify cells between the G0/G1 and G2/M peaks as being in S phase of DNA replication.
6.G2/M peak - Draw a gate around the second peak of cells with 4N DNA content (G2/M phase).
7.Determination of percentages - Use gating and statistics tools to determine the percentage of cells in each phase of the cell cycle (G0/G1, S, G2/M).
The key steps are removing doublets and debris, then gating on the DNA content peaks to classify cells into their respective phases based on their DNA ploidy. Proper controls and compensation are also important for accurate cell cycle analysis.

☒ Tick this box to confirm that a figure exemplifying the gating strategy is provided in the Supplementary Information.
