# Supplementary figures and images for: mTORC2-driven chromatin cGAS mediates chemoresistance through epigenetic reprogramming in colorectal cancer
Source: Nat Cell Biol. 2024 Jul 30;26(9):1585–96. doi: 10.1038/s41556-024-01473-0 (PMC11392818; doi:10.1038/s41556-024-01473-0)

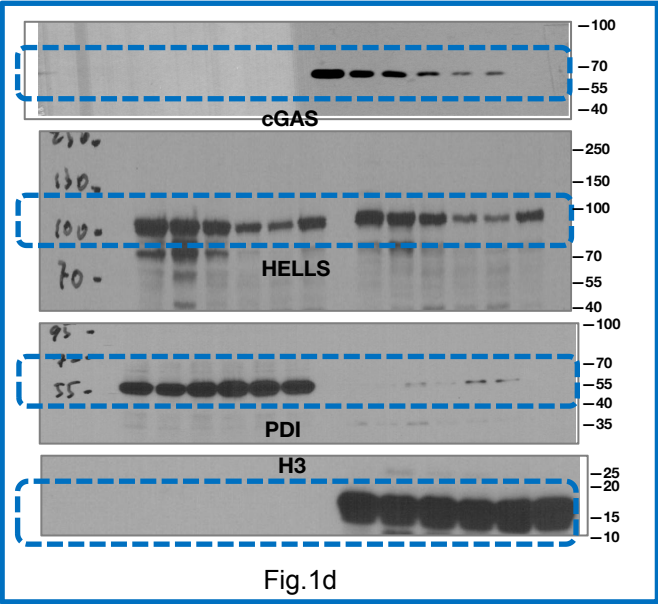

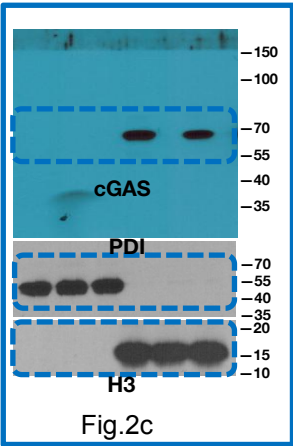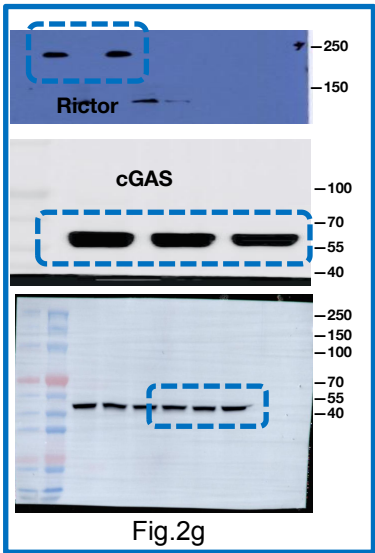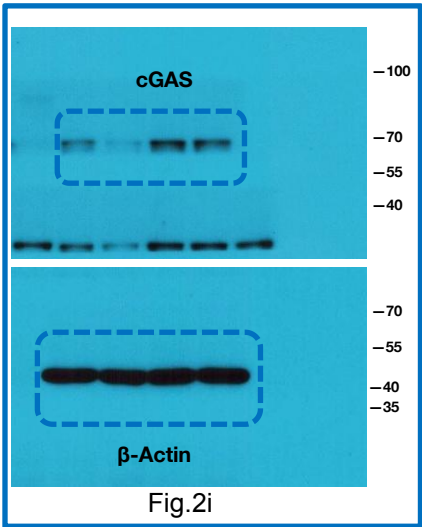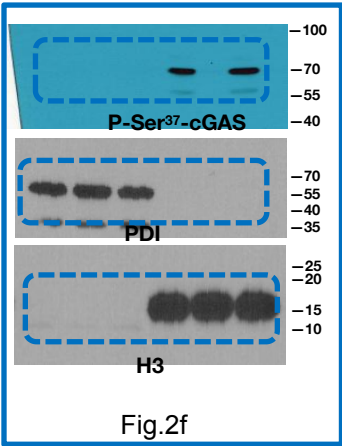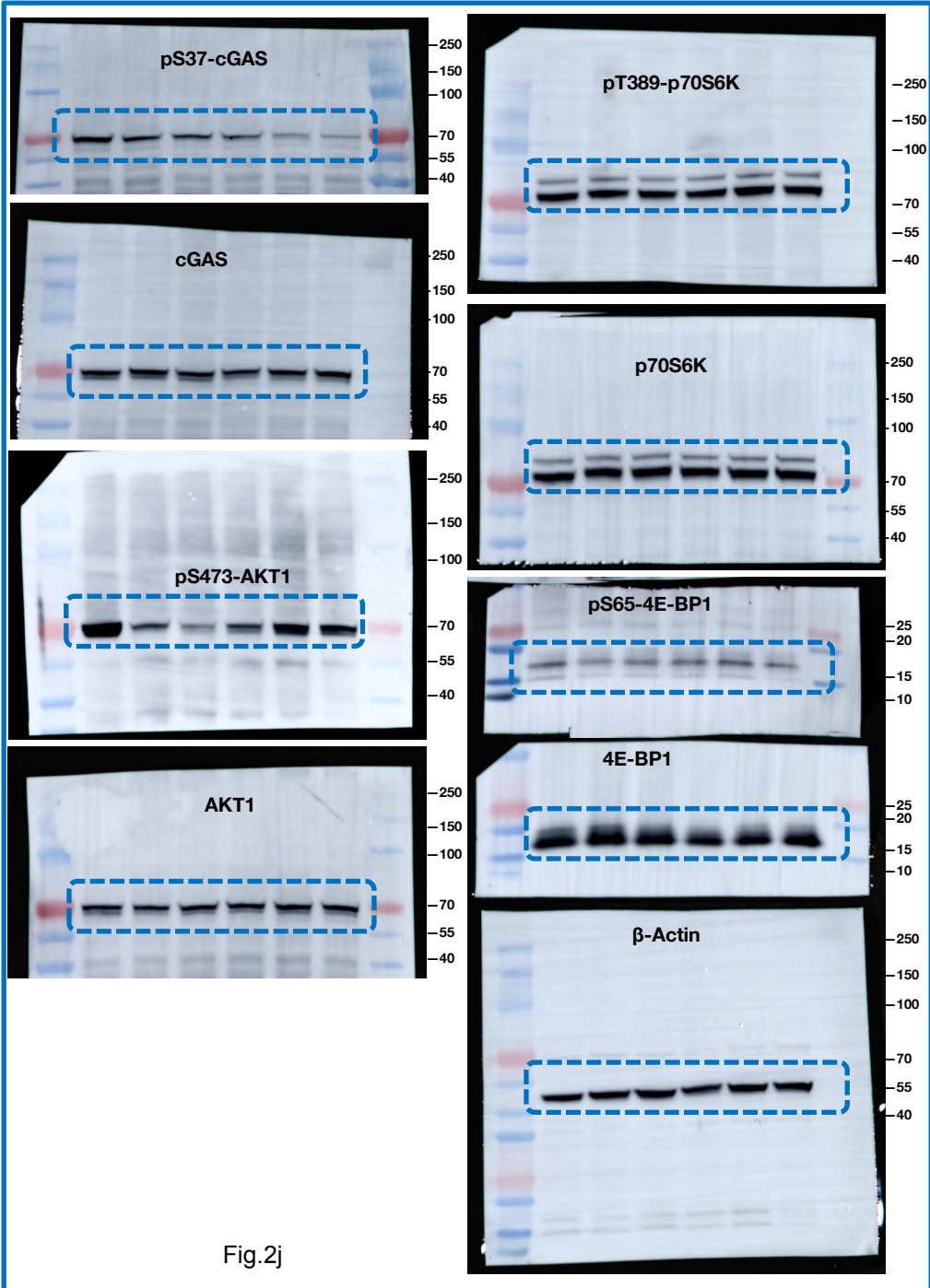

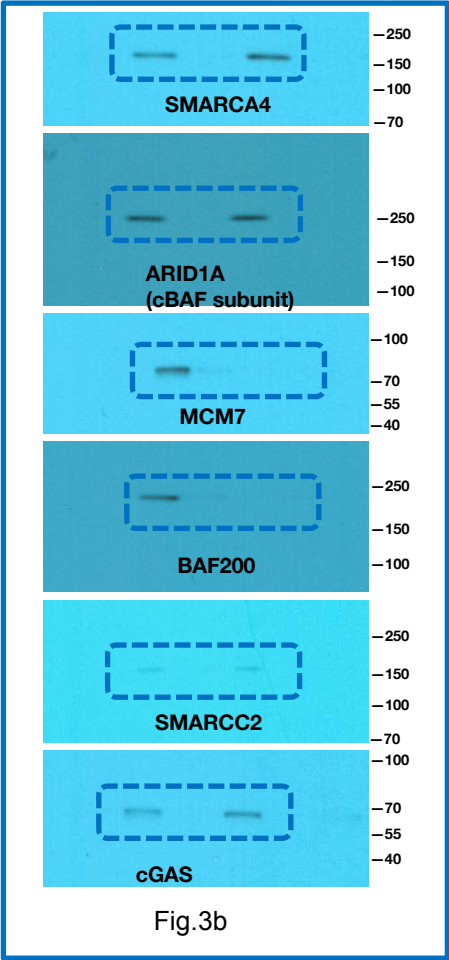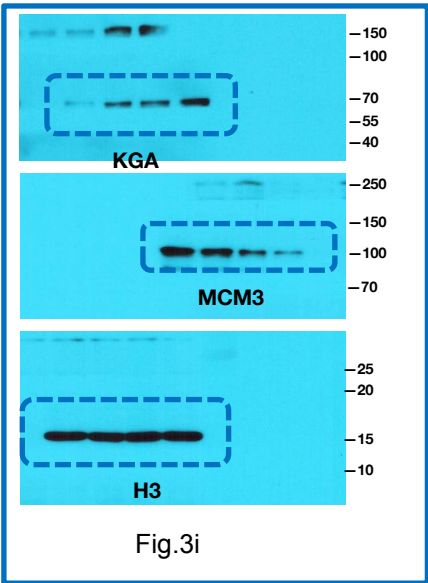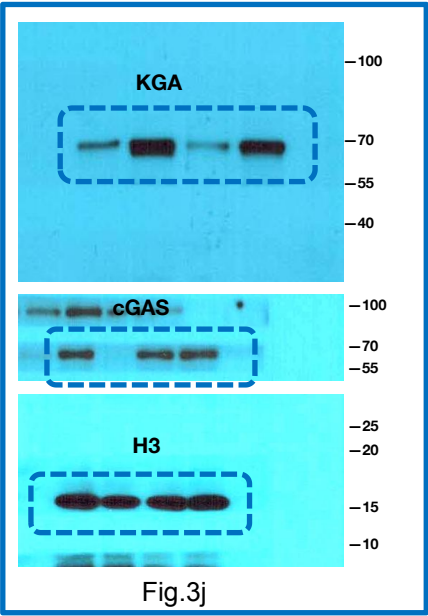

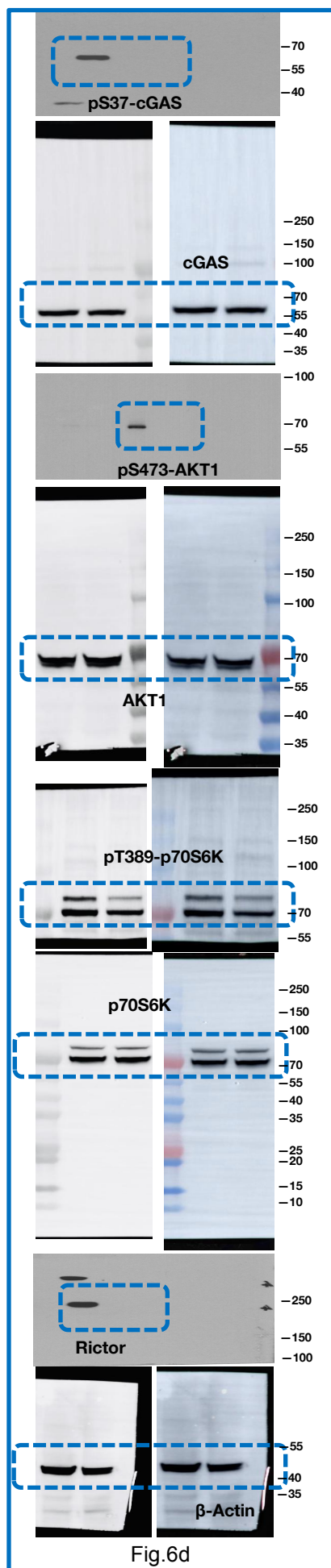

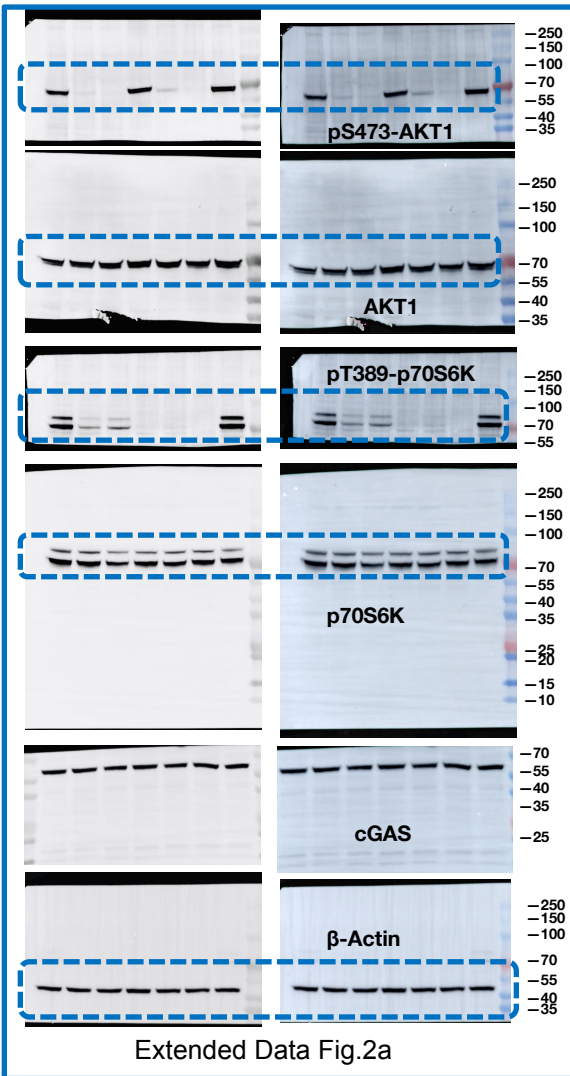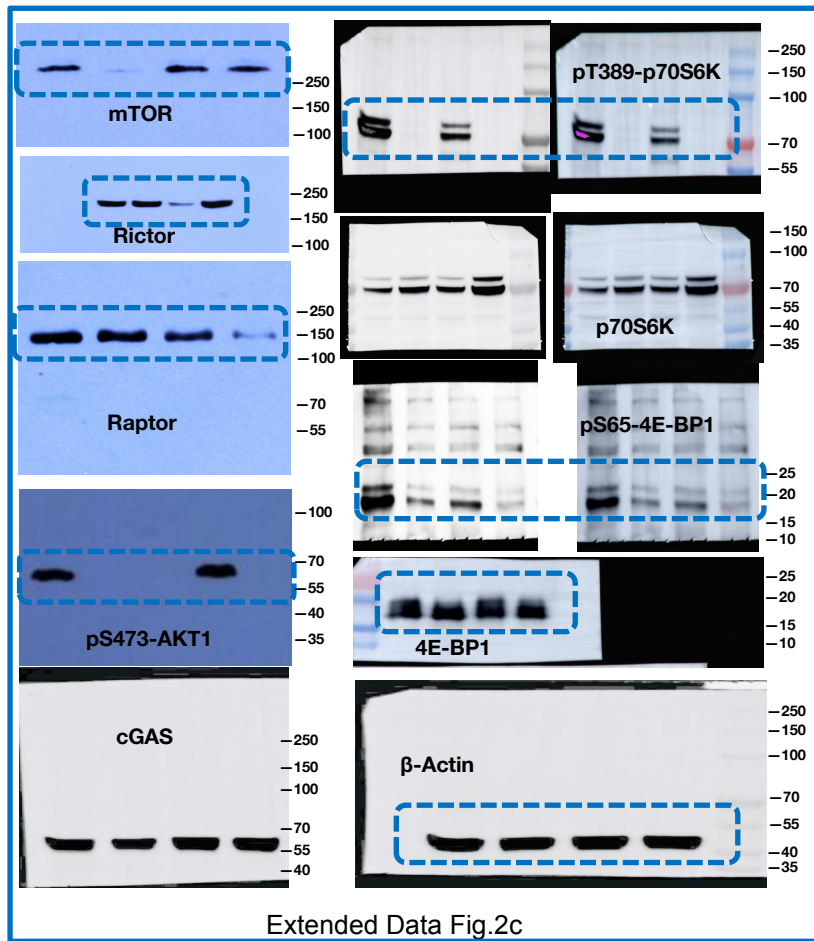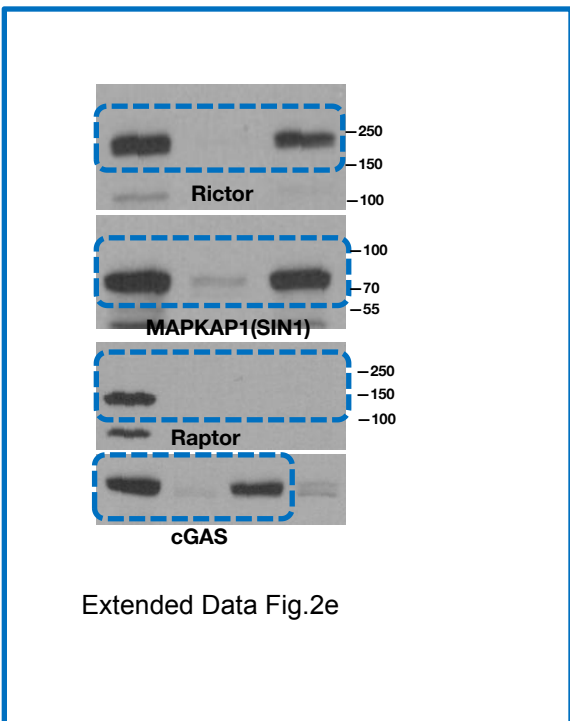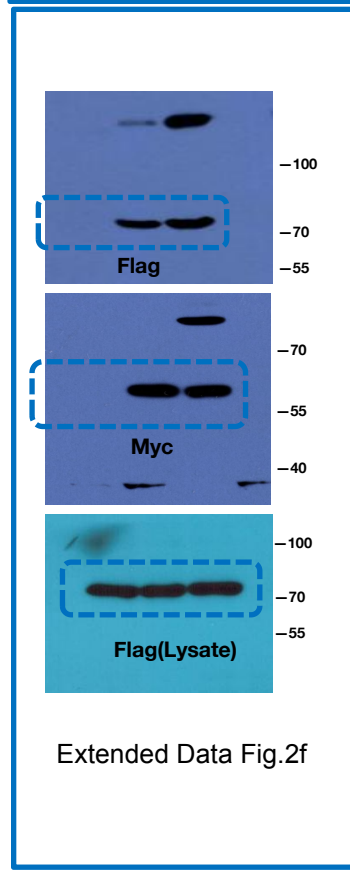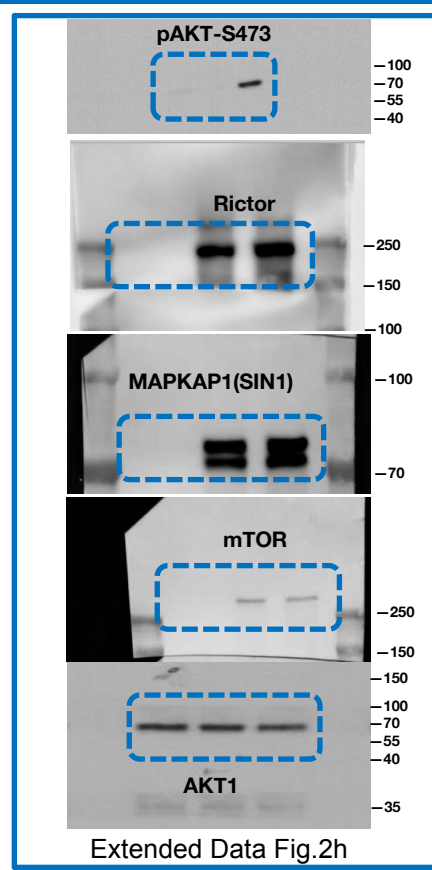

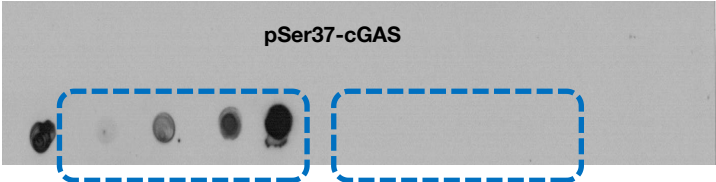

Extended Data Fig.3d

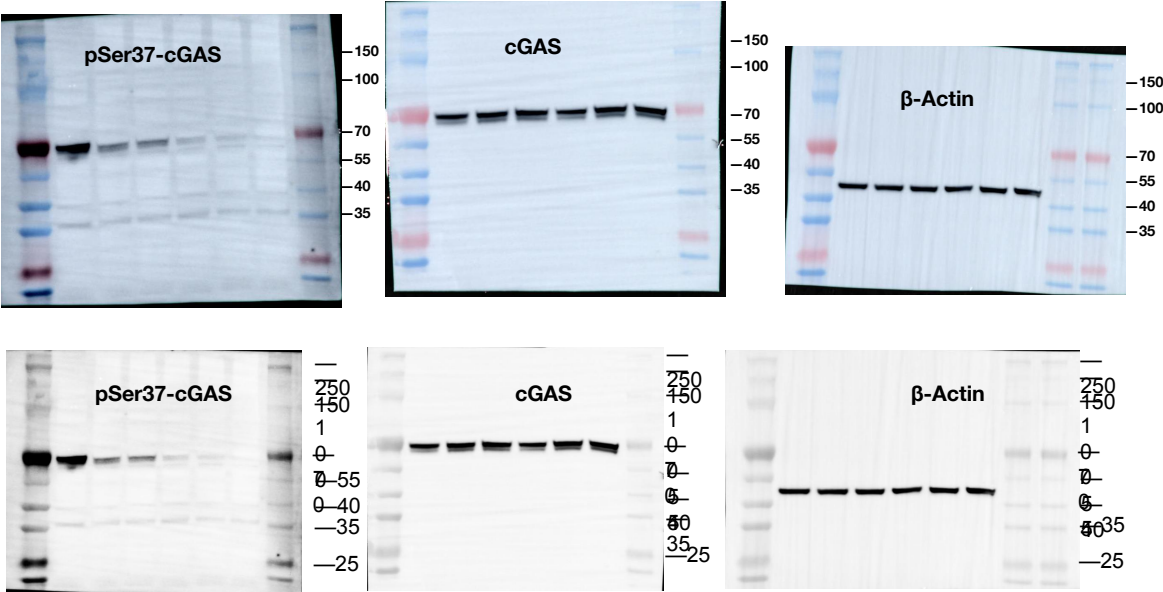

Extended Data Fig. 3e

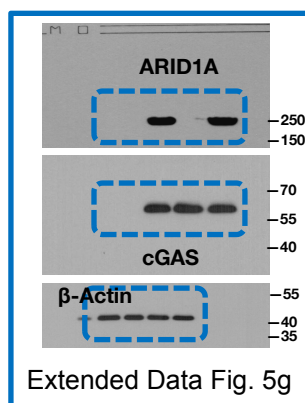

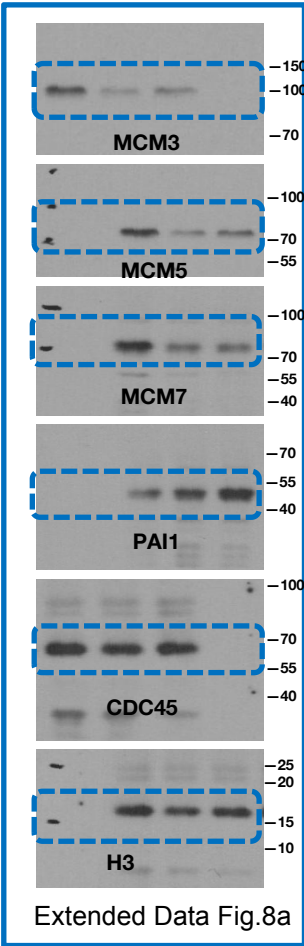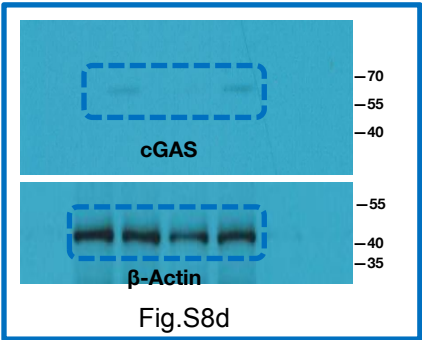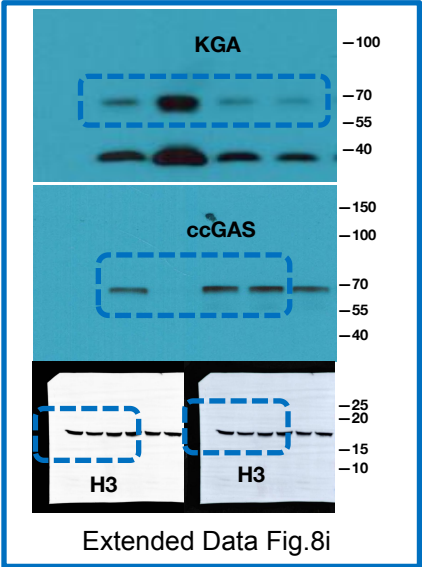

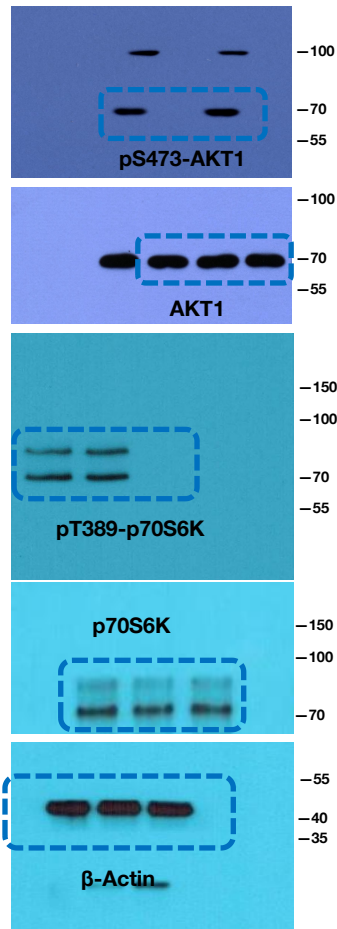

Extended Data Fig.10b

Supplement: Supplementary file 10 — Unprocessed western blots and/or gels. Statistical source data. [file 41556_2024_1473_MOESM10_ESM.pdf]
